# Estimating Operational Costs of Activated Carbon for Water Treatment Plants by Predicting the Rise of Harmful Algal Blooms Under Climate Change in Korea Using Machine Learning

**DOI:** 10.1002/wer.70310

**Published:** 2026-03-10

**Authors:** Jayun Kim, Himchan Park, John J. Lenhart, Jiyoung Lee, Kendall Byrd, Gayeon Jang, Sangjun Kim, Joonhong Park

**Affiliations:** ^1^ Department of Civil and Environmental Engineering Yonsei University Seoul The Republic of Korea; ^2^ Division of Environmental Health Sciences, College of Public Health The Ohio State University Columbus Ohio USA; ^3^ Department of Intelligent Data and Optimization Yonsei University Seoul The Republic of Korea; ^4^ Department of Civil, Environmental and Geodetic Engineering The Ohio State University Columbus Ohio USA; ^5^ Department of Food Science & Technology The Ohio State University Columbus Ohio USA

**Keywords:** cost analysis, cyanobacteria, data‐driven model, global warming, microcystin, T&O compounds

## Abstract

The escalating frequency of harmful cyanobacterial blooms (HCBs), driven by climate change and eutrophication, poses risks to ecosystems, water resources, and public health. Given South Korea's heavy reliance on surface waters, increasingly affected by HCBs producing microcystins and taste and odor compounds (geosmin and 2‐methylisoborneol), this study used machine learning to predict cyanobacterial proliferation by 2100 under climate scenarios. It also estimates increases in treatment costs, assuming water treatment plants (WTPs) respond to increased bloom intensity solely by modifying their usage of powdered activated carbon (PAC). A random forest (RF) model trained on 28 years of Nakdong River data projected HCB occurrences under Shared Socioeconomic Pathway 5–8.5. The RF indicated significant increases in HCB magnitude and variability (cyanobacteria densities from 1.6 × 10^4^ to 6.3 × 10^4^ cells/mL; coefficient of variation from 1.60 to 1.77), corresponding to a 6.7°C increase in mean annual air temperature. Analysis of WTP operational data and prior studies revealed a correlation between PAC use and HCB events, suggesting the increase in HCBs necessitates significantly higher PAC doses to treat projected secondary metabolites, particularly microcystins. Under the worst‐case scenario, the projected cost burden for water treatment could triple from current levels, potentially reaching $22.1/month/household by 2100, supporting proactive implementation of advanced treatment facilities in high‐risk regions. These findings underscore the need for enhanced preparedness to address more complex HCB patterns under climate change, ensuring water safety, economic stability, and human health. Additionally, this study provides a methodological blueprint for other countries facing similar climatic and environmental challenges.

## Summary

- Investigating future operational costs in water treatment (WT) facilities.
- Random forest projected 3–5 times more abundant cyanobacteria by 2100 (SSP5–8.5).
- Increase in microcystins demanded substantially more powdered activated carbon.
- WT costs to rise from $3–12 to $6–22/household/month under SSP5–8.5.
- Proactive measures are necessary to mitigate health risks and WT burdens.

## Graphical abstract

This study investigated future operational costs in water treatment (WT) plants. Random forest modeling projected 3–5 times more abundant cyanobacteria by 2100 due to climate change. Increased microcystins demanded substantially more activated carbon and, as a result, WT costs could rise to $6–22/household/month. Proactive measures are necessary to mitigate health risks and WT burdens.

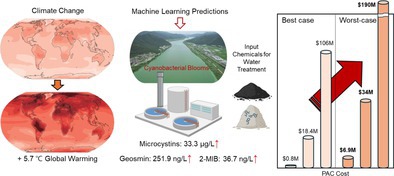

## 1 Introduction

In recent decades, the frequency of harmful algal blooms (HABs) in surface waters around the globe has significantly increased, largely due to climate change and increased eutrophication (Heisler et al. 2008; Taranu et al. 2012; Hou et al. 2022). The toxins released during these blooms not only negatively impact ecosystems, but also jeopardize water resources used for drinking water sources, recreation, and food production (Hitzfeld et al. 2000; Lee et al. 2019). Recent studies have demonstrated that cyanobacterial blooms contribute significantly to cyanotoxin contamination in freshwater, posing additional risks to human health and destabilizing aquatic ecosystems (Ahmad et al. 2025). This underscores the necessity for robust prediction and treatment tools at drinking water facilities (Ma et al. 2023). Most of South Korea's (hereafter referred to as Korea) water supply derives from surface water sources, such as rivers and reservoirs (Ministry of Environment 2021). Consequently, mitigating the impact of HABs on the safety of water resources is an urgent national environmental priority, particularly in the Nakdong River Basin, which experiences severe HABs annually (Lee et al. 2019; Park et al. 2018).

Cyanobacterial blooms are particularly concerning among freshwater HABs because certain cyanobacteria can produce taste and odor (T&O) compounds such as geosmin and 2‐methylisoborneol (2‐MIB), as well as cyanotoxins. T&O events have attracted considerable attention from water utilities and researchers around the globe, with numerous cases involving these two odorants reported over the years (Devi et al. 2021). Although not a health risk, these T&O compounds greatly impact the aesthetic appeal of drinking and recreational water. Consequently, this can reduce the public's confidence in the safety of the water. Among the various identified odorants, geosmin and 2‐MIB, which impart a musty or earthy odor, have been extensively studied (Bruchet 2019; Lee et al. 2017; Watson et al. 2016). Both compounds exhibit very low odor threshold values (< 10 ng/L), making them difficult to remove effectively with conventional water treatment processes (Lu et al. 2019; Tsao et al. 2014). Cyanotoxins, such as microcystins (MCs), directly affect the health of humans, animals, and plants (Zhang et al. 2015; Pham and Utsumi 2018). Additionally, people living near HAB‐affected water bodies may have higher rates of acute and chronic liver diseases (Lee et al. 2019; Gorham et al. 2020) and other negative health outcomes (Lee, Choi, et al. 2022). For example, prior research has suggested that prolonged consumption of tap water containing small amounts of MCs could potentially increase the risk of liver cancer and colorectal cancer (Martínez Hernández et al. 2009). Therefore, it is crucial to accurately predict harmful cyanobacterial blooms (HCBs) that produce cyanotoxins and T&O compounds to minimize health risks and optimize water treatment processes.

Conventional water treatment processes are ineffective at removing secondary metabolites of cyanobacteria in source water, necessitating the addition of supplemental treatment processes (Westrick et al. 2010). Among various treatment methods, powdered activated carbon (PAC) is commonly used to effectively reduce the risk from HCBs in drinking water due to its low cost, ease of use, and limited requirements for supplemental infrastructure (He et al. 2016). These features have resulted in PAC being used in water treatment processes to treat a variety of organic compounds (Park et al. 2021; Cook et al. 2001; Bertone et al. 2018; Ho et al. 2011) since the early 1900s (Çeçen and Aktaş 2011). Pre‐oxidation or pre‐disinfection processes are an option to PAC (Cheung et al. 2013), but they come with high capital costs, increased energy demands, and potential side effects, such as the formation of trihalomethanes and other disinfection byproducts (DBPs) as observed in studies of the western Lake Erie region (Lee et al. 2023). Furthermore, cell lysis may occur, leading to increased extracellular toxins as well as T&O compounds (He and Wert 2016). This could subsequently result in the need to add further supplemental processes. Under scenarios with increased HCBs, increased use of PAC could serve to effectively reduce the potential increase in secondary metabolites of cyanobacteria and limit the formation of DBPs (Zheng et al. 2023).

The cost of PAC treatment varies, but in the United States it can be approximately $19 per person per year (system size 3800 m^3^/d, current value) (Najm et al. 1991). This corresponds to roughly 10% of total water costs, averaging about $186 per person per year in the US (Unger et al. 2023; US EPA 2025). The production cost for tap water in Korea is $63 per person per year, which is 39% of the global average, due to a very high revenue‐water ratio (96%; Seoul Special Metropolitan City 2024; Global Water Intelligence 2023). In Korea, water treatment facilities commonly use PAC to remove trace organics, such as those produced by HCBs, typically at concentrations of 5–30 mg/L (Kim and Choi 2015; Jeong et al. 2017; Park et al. 2019). Because Korea relies on imports of activated carbon, the government recently designated it as one of the three emergency strategic commodity items (Korean Ministry of Economy and Finance 2022). Despite the low initial cost for PAC, this study hypothesizes that chemical costs in water treatment utility operations are non‐negligible and must be adjusted according to MC and T&O compound levels in source waters associated with HCB occurrence. It has been reported that a decline in water quality over the past century resulted in an approximately 53% annual increase in operation and maintenance costs for water treatment plants (WTPs) (McDonald et al. 2016). Therefore, to optimize costs and resources in water treatment, it is essential to evaluate PAC requirements under varying source water conditions for efficient operation and management.

Currently, there is a growing body of research focusing on proactive responses to HCBs by predicting their occurrence in the short‐ and mid‐term using machine learning (Kim, Jung, et al. 2023; Wang et al. 2024). Additionally, machine learning approaches have been applied to project long‐term algal blooms due to climate change (Ma et al. 2025). These predictions utilize extensive datasets that include environmental variables associated with HCBs, with temperature (both air and water) being one of the most influential factors. Elevated temperatures not only directly accelerate the proliferation of cyanobacteria (Cha et al. 2017; Kim et al. 2024) but also enhance stratification of the water body, which further stimulates the occurrence of HCBs (Jöhnk et al. 2008; Paerl and Huisman 2008). HCBs are anticipated to worsen due to climate change (Ralston and Moore 2020; Paerl and Barnard 2020). Increases in HCB intensity will likely necessitate enhanced use of activated carbon and other treatment approaches to mitigate potential adverse impacts. However, few studies have investigated the long‐term cost implications for WTPs using long‐term projections of HCBs. Therefore, in an era of changing climate, it is essential to understand how future changes in HCBs could impact management of water resources by projecting operational costs for water treatment based on anticipated temperature increases.

In this study, reported data and machine learning were used to project HCBs and estimate future PAC costs, with the objectives to: (1) investigate the operational costs and usage of PAC in WTPs, (2) predict cyanobacteria proliferation under climate warming scenarios based on Shared Socio‐economic Pathways (SSPs), and (3) estimate the future costs of PAC needed to mitigate HCBs. To achieve these objectives, data on water treatment operations provided by Korean WTPs were analyzed, and machine learning modeling was employed, using SSPs and environmental data collected along the Nakdong River, to predict long‐term cyanobacteria trends. PAC dosages and costs were estimated to treat T&O compounds and MCs produced by the projected HCBs, using PAC‐removal rate relationships derived from published experimental results. We anticipate the findings of this study could be used by WTP operators to enhance preparedness for climate change, and the methodologies developed can be adopted for use in other countries.

## 2 Materials and Methods

### 2.1 Data Acquisition and Study Sites

Data used in this study were accessed from annual reports on water supply and management practices issued by the Korean government agency K‐water from 2011 to 2022 (https://www.kwater.or.kr/gov3/sub03/publicList.do?s_mid=1664). Details of the operating conditions of the 38 WTPs which K‐water manages were retrieved to extract data on the monthly amount of water treated, total chemical usage, PAC usage, and PAC cost. The location of each WTP and its corresponding water source were identified, and cyanobacteria abundance data from 52 cyanobacteria alert sites were acquired from Korea's Water Environment Information System (WEIS) (http://water.nier.go.kr/web/algaeStat?pMENU_NO=195), operated by the National Institute of Environmental Research. Cyanobacterial data were selected because they were more extensively available than other indicators associated with health risk and water treatment burden, enabling feasible and reliable subsequent modeling and cost analysis. These data were then used to compare the operations of WTPs with HCB occurrences in proximity to the water source. Monthly and annual averages of these cyanobacteria data (microscopic counting and identification) were compared with the monthly WTP operational statistics.

**FIGURE 1:**
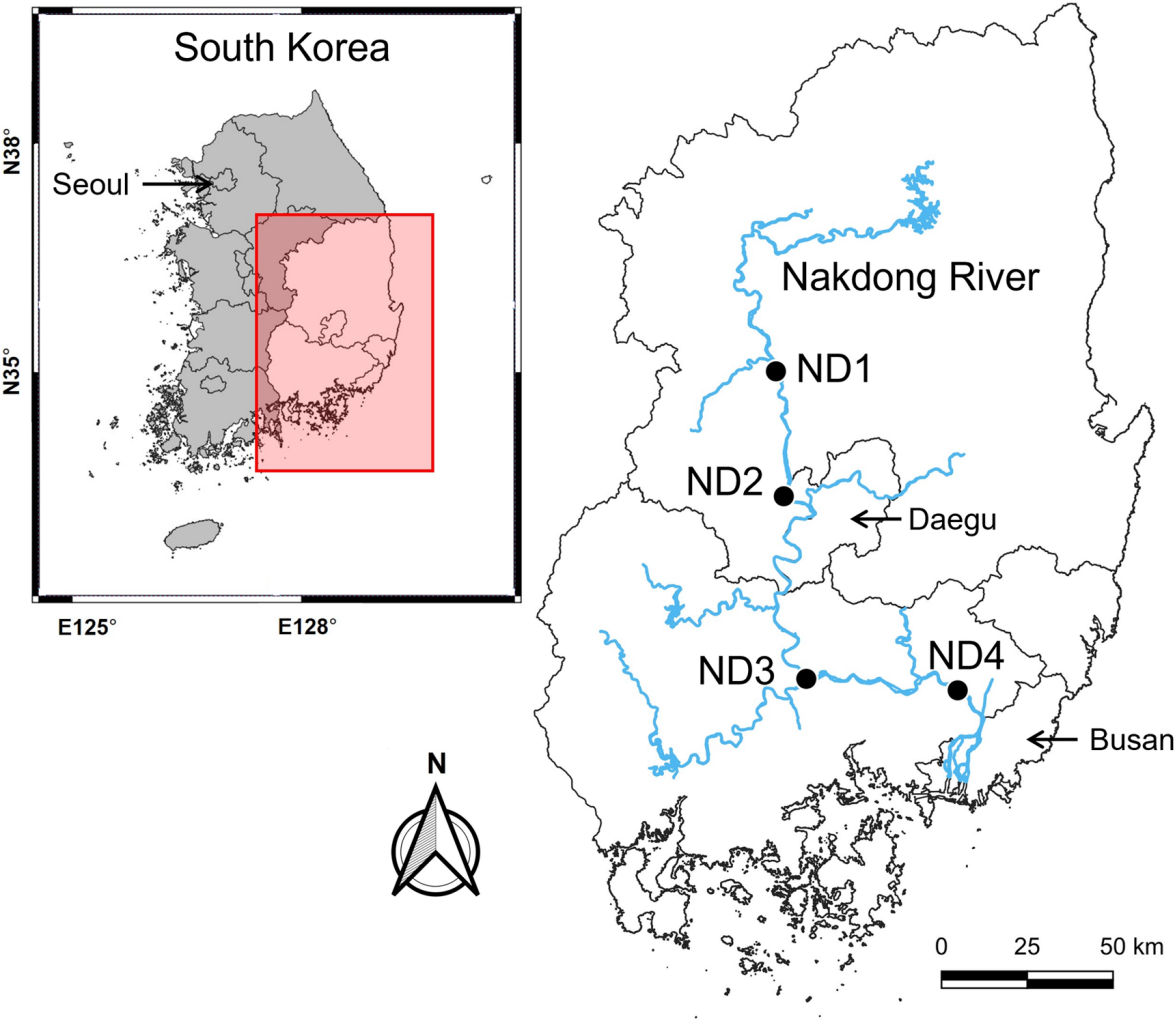
Map illustrating locations of the harmful algal bloom alert sites ND1–ND4 along the Nakdong River corresponding to locations where water quality data were collected.

Data used in the machine learning modeling were retrieved from WEIS, which compiles data collected by local government agencies. Data for four HCB alert sites along the Nakdong River identified by WEIS as “drinking water sources” were selected (Figure 1, ND1 to ND4, from upstream to downstream). Weekly data on cyanobacteria density (or twice a week when cyanobacteria ≥ 10,000 cells/mL) and water quality parameters were collected during 2016–2023 at ND1–3 and 2020–2023 at ND4 (with the start year indicating the first year of observation at each site). The water quality parameters include biochemical oxygen demand (BOD, mg/L), chlorophyll‐*a* (Chl‐a, mg/m^3^), chemical oxygen demand (COD, mg/L), dissolved oxygen (DO, mg/L), electrical conductivity (EC, μS/cm), pH, suspended solids (SS, mg/L), total nitrogen (TN, mg/L), total phosphorus (TP, mg/L), and water temperature (°C). Additionally, water velocity (m/s) was calculated at each site with daily flow rate data using site‐specific empirical flow rate‐water velocity equations (Table S1; Kim et al. 2026). Air temperature at the study sites was estimated from measurements taken at weather monitoring stations located near the four HCB alert sites that are operated by the Korea Meteorological Administration (KMA).

### 2.2 Prediction of Future Cyanobacteria Density

A random forest (RF) model was used to predict cyanobacteria density. RF is a machine learning algorithm comprising multiple decision trees (Breiman 2001). The RF model was trained for each study site using 11 independent variables (BOD, Chl‐a, COD, DO, EC, pH, SS, TN, TP, water temperature, and water velocity) and one dependent variable (cyanobacteria in log cells/mL). Data from December to March were omitted because HCBs rarely occur in this period. Additionally, data sets with any missing values among the independent variables or cyanobacteria density = 0 cells/mL were excluded. Model performance was validated using the Nash‐Sutcliffe model efficiency coefficient (NSE; Nash and Sutcliffe 1970) and mean‐squared error (MSE; Fournier et al. 2024), employing leave‐one‐out cross‐validation (Gronewold et al. 2009). Leave‐one‐out cross‐validation involves using the entire dataset, except for one data point, to train the model; the excluded data point is then used to validate the model. This process is repeated for each data point in the dataset. As a result, this method produces a robust validation performance, reflecting the reliability of the use of the model for long‐term predictions, by using all the data as validation data. Machine learning modeling was implemented using the Scikit‐learn library and the Python language.

Estimates of the future air temperature at the study sites were used to simulate the future environment. This study adopted SSP5–8.5, a worst‐case climate change scenario, for the future air temperatures. SSP5–8.5 assumes continued and substantial reliance on fossil fuels and underscores indiscriminate, city‐centered development aimed at the rapid advancement of industrial technology. This scenario, established by the Intergovernmental Panel on Climate Change (IPCC), represents very high greenhouse gas emissions and projects a global mean air temperature increase of 3.3–5.7°C by 2100 relative to 1850–1900 (IPCC 2021). For long‐term predictions, temperatures for the study sites in 2091–2100 estimated under SSP5–8.5 were obtained from the Climate Information Portal (http://www.climate.go.kr/home/) operated by the government agency, KMA. Data on climate change were provided by employing local climate projections derived from global climate models (KMA Advanced Community Earth System Model and UK Earth System Model 1) and East Asia climate models (Kim, Min, et al. 2023). Under the SSP5–8.5 scenario, projected mean annual air temperatures for 2091–2100 at sites ND1, ND2, ND3, and ND4 were 19.7°C, 19.8°C, 20.1°C, and 21.0°C, respectively, compared with 12.9°C, 13.0°C, 13.4°C, and 14.4°C during 2000–2019.

Future cyanobacteria densities under SSP5–8.5 were predicted using the trained RF model and projected increases in air temperature. We first calculated the temperature rise at each site by comparing observed air temperatures with projected monthly temperatures for 2091–2100. These air temperature increases were then correlated with water temperatures through linear regression analysis (water temperature (°C) = 0.881 × air temperature (°C) + 4.302, *R* ^2^ = 0.90). The resulting elevated water temperatures were put into the trained model for each site. Subsequently, a proportional relationship was established between outputs of the model using observed data and data with adjusted water temperatures. To minimize bias due to rare‐event data (i.e., events with water temperatures exceeding 26.7°C, 28.6°C, 29.4°C, and 29.2°C at ND1–4, respectively), only data with water temperatures less than or equal to the 75th percentile of the observed dataset were considered. Using this proportional relationship, current predicted cyanobacteria densities were transformed into future predicted densities. Prediction results for the future environment under SSP5–8.5 were compared with the prediction results for current environment.

### 2.3 Determination of PAC Dose and Future Cost Estimation

To estimate the PAC dosage, geosmin, 2‐MIB, MCs, and MC‐LR concentrations were first determined. This was accomplished using previous studies to establish relationships for converting cyanobacteria density values (cells/mL) to dissolved geosmin (Tsao et al. 2014), 2‐MIB (Chiu et al. 2016; Chiu et al. 2017), and MCs (Fitzgerald et al. 1999; World Health Organization [WHO] 2003; Health Canada 2022). These relationships were then used to convert the predicted cyanobacteria densities to the corresponding compound concentration. Additional relationships between cyanobacteria densities and concentrations of geosmin, 2‐MIB, and MC‐LR (both intracellular and extracellular) in the Nakdong River study site were examined through regression analysis to facilitate conversion from cyanobacteria densities to geosmin, 2‐MIB, and MC‐LR levels. This resulted in two relationships for geosmin, two for 2‐MIB, one for total MCs, and one for MC‐LR. The values of geosmin and 2‐MIB from these four relationships were determined and averaged to estimate the concentration of T&O compounds.

Empirical models were established using previously published data to estimate the necessary PAC dose required for effective removal of these compounds (Table S2). For removing T&O compounds, this study developed a model (the “MoE Model”) using data from the Korean Ministry of Environment (MoE; Ministry of Environment and National Institute of Environmental Research 2017), describing relationships between PAC doses and residual concentrations of geosmin and 2‐MIB. For MCs removal, the “Ho Model” was developed based on the findings of Ho et al. (2011), and for MC‐LR removal, the “EPA Model” was developed based on results published by the US Environmental Protection Agency (US EPA 2016) and Bajracharya et al. (2019). These three models demonstrate the removal of T&O compounds, MCs, and MC‐LR, respectively, relative to PAC doses. Thresholds for acceptable levels of T&O compounds, MCs, and MC‐LR in treated water were set at 20 ng/L, 1.6 μg/L, and 1.0 μg/L, respectively, in accordance with domestic (MoE) and international guidelines (Young et al. 1996; WHO 2022; US EPA 2015). The PAC doses needed to reduce the future concentrations of the three compounds to the established thresholds were subsequently determined for both worst‐case (SSP5–8.5) and best‐case (assuming current HCB patterns persist) scenarios. Based on these doses, the required monthly amount of PAC (in kg) for adequate treatment of T&O compounds, MCs, and MC‐LR concentrations in raw water during HCB season was calculated for each study site (ND1–4). This calculation considered the daily water treatment capacity at the respective WTP. Subsequently, the estimated PAC usage was converted into PAC cost, taking into account the service population. The Korean Won to US$ conversion factor (1166 KRW = 1 USD, the mean currency exchange rate from 2014 to 2023) and the mean annual inflation rate of 2.52% during 2001–2023 were used to estimate future costs.

## 3 Results and Discussion

### 3.1 Current State of WTP Operations

**FIGURE 2:**
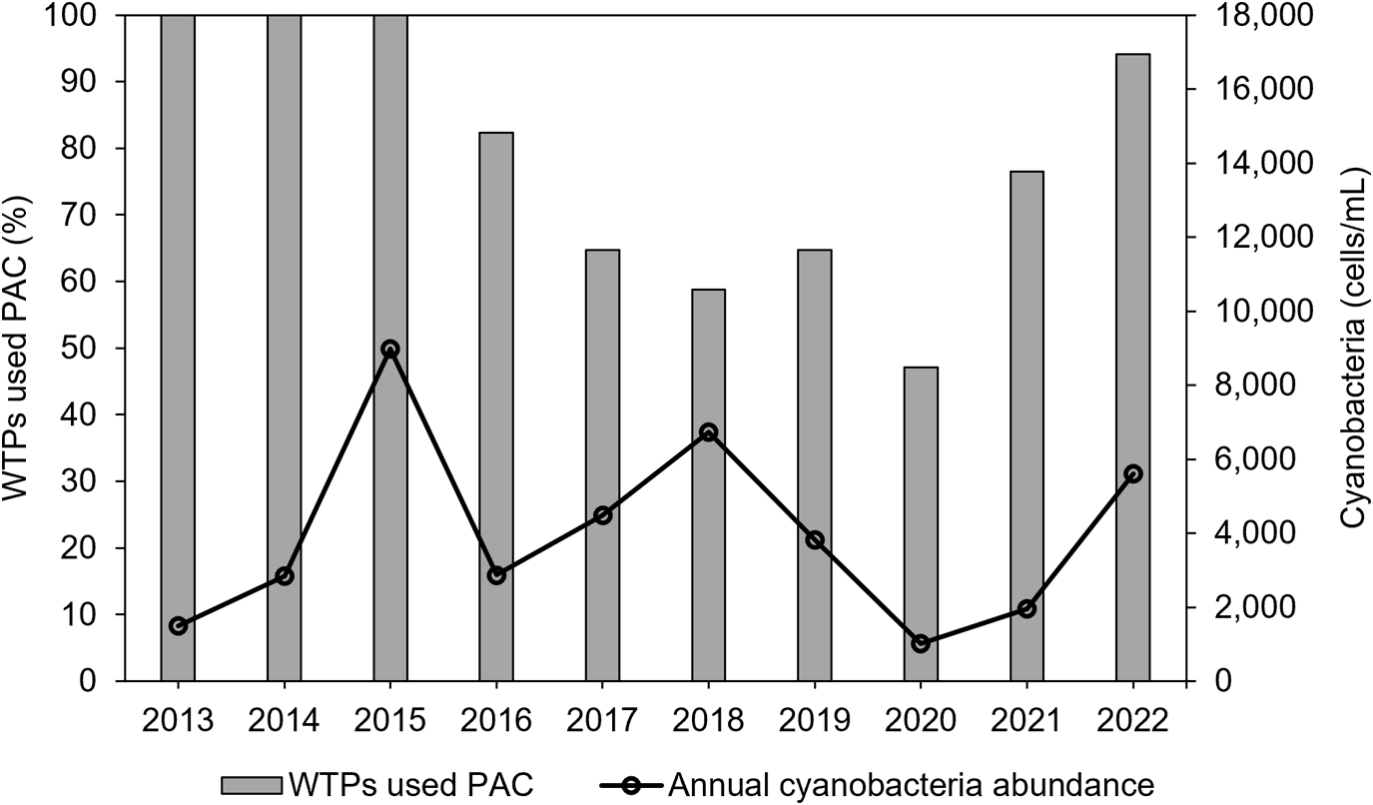
For a total of 19 water treatment plants (WTPs), the annual proportion of WTPs that used powdered activated carbon (PAC; left axis) and nationwide mean cyanobacteria abundance (right axis) is shown.

The majority of the WTP intakes in Korea (93.9%) rely on surface waters, such as rivers and reservoirs, across the country (Figure S1). Additional intake sources include riverbeds and groundwater. With such a high use of surface water it is clear that HCBs are closely linked to public water consumption, emphasizing the critical need for effective surface water quality management. Out of the 38 WTP study sites, 19 indicate using PAC in their water treatment processes in their publicly available annual reports. In general, the number of WTPs utilizing PAC decreases in years with milder HCBs (2015–2016 and 2019–2020) and increases in years when annual HCBs were more severe (2020–2022) (Figure 2). This trend highlights the importance of predicting PAC requirements in response to escalating HCBs.

**FIGURE 3:**
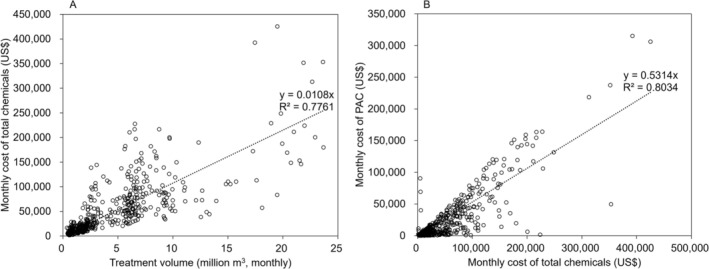
Relationships of (A) water treatment volume and chemical costs and (B) chemical costs and powdered activated carbon (PAC) costs.

The relationships between chemical costs, treatment volume, and PAC costs were examined (Figure 3). As expected, the use of chemicals (including PAC, polyaluminum chloride, polyamines, polyaluminum silicate chloride, sodium hypochlorite, and hydrogen peroxide) increased linearly with the volume of water treated. On average, approximately $160,000 is spent to treat 15 million m^3^ of water, supplying 1 million people monthly, as observed in this study (Figure 3A). PAC alone accounts for approximately 53% of the chemical costs (Figure 3B), indicating its critical role and need for prioritizing its management for the effective operation of WTPs. This equates to $0.2 per month for a single household.

**FIGURE 4:**
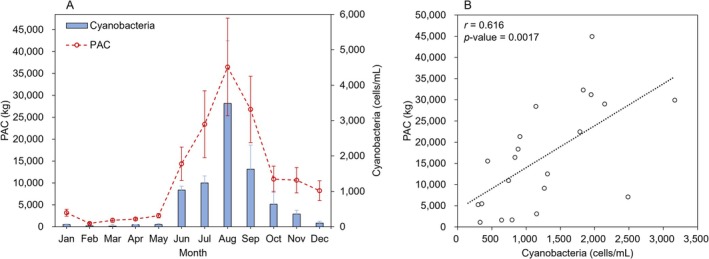
Comparison of powdered activated carbon (PAC) use in kg/month with HCB occurrence (cells/mL). (A) Average monthly PAC usage across the 19 WTPs utilizing PAC and mean national cyanobacterial cell counts (error bars indicate one standard error). (B) Comparison of PAC usage and cell counts from June to September, representing months of peak HCB occurrence (dotted line is a regression plot).

Using data available from WEIS, PAC usage at the 19 WTP study sites was compared with the magnitude of HCB occurrence (Figure 4). The results indicate that PAC usage was higher during summer months than during winter months (Figure 4A). PAC usage drastically increased from May to June, coinciding with higher cyanobacteria densities, peaked in August when HCB levels were highest, and then decreased. The average PAC usage across the study sites in August was approximately 36,500 kg per WTP or 5.6 mg/L. Based on the conversations with WTP operators, this value is within the typical range of PAC dosages used during bloom season. The amount of PAC used in the study sites demonstrated a significant correlation (*p*‐value < 0.01) with the abundance of cyanobacteria in the source water (Figure 4B). This implies that the WTPs likely adjusted their PAC input in response to the level of HCBs during the summer months.

### 3.2 Future HCB Simulation

Using cyanobacteria density, water‐quality, and water‐temperature data, RF models were trained for ND1, ND2, ND3, and ND4 along the Nakdong River. The model training performances had NSE values greater than 0.95 at all sites, while validation NSEs were 0.382, 0.537, 0.589, and 0.670 with corresponding MSEs of 0.255, 0.375, 0.396, and 0.312 at ND1, ND2, ND3, and ND4, respectively, which are within an acceptable range. These error rates indicate an uncertainty range in the prediction outputs, suggesting mean potential underestimations of 6.7 × 10^2^ and 5.5 × 10^3^ cells/mL compared to actual mean cyanobacteria densities of 2.2 × 10^3^ and 2.0 × 10^4^ cells/mL at sites ND1 and ND4 (representing the minimum and maximum mean densities, respectively). The relatively low NSE at ND1, despite its low MSE, reflects the site's low magnitude and limited variability in cyanobacterial density. Because NSE is normalized by observed variance, small absolute errors can still yield low NSE. By contrast, ND4 showed the best performance despite the smaller dataset size, likely because the broader and more evenly distributed range of cyanobacterial densities provided a stronger signal for model learning.

Water temperature emerged as a critical variable for predicting cyanobacteria at all sites (Figure S2), consistent with previous work (Kim et al. 2021). The second most important variable was TN, which may be attributed to its negative correlation with water temperature, with correlation coefficients of −0.36, −0.42, −0.56, and −0.61 at ND1, ND2, ND3, and ND4, respectively. This aligns with previous findings showing negative correlations between nitrogen parameters and MC (Buley et al. 2022), potentially attributable to enhanced denitrification at higher temperatures (Hu et al. 2022). Recent advances in explainable artificial intelligence, such as Shapley Additive exPlanations and multi‐run class activation mapping, have enabled robust identification of key drivers of algal blooms (Lee, Kim, et al. 2022; Lee and Jeon 2025), thereby improving model interpretability for policy‐relevant decision‐making.

**FIGURE 5:**
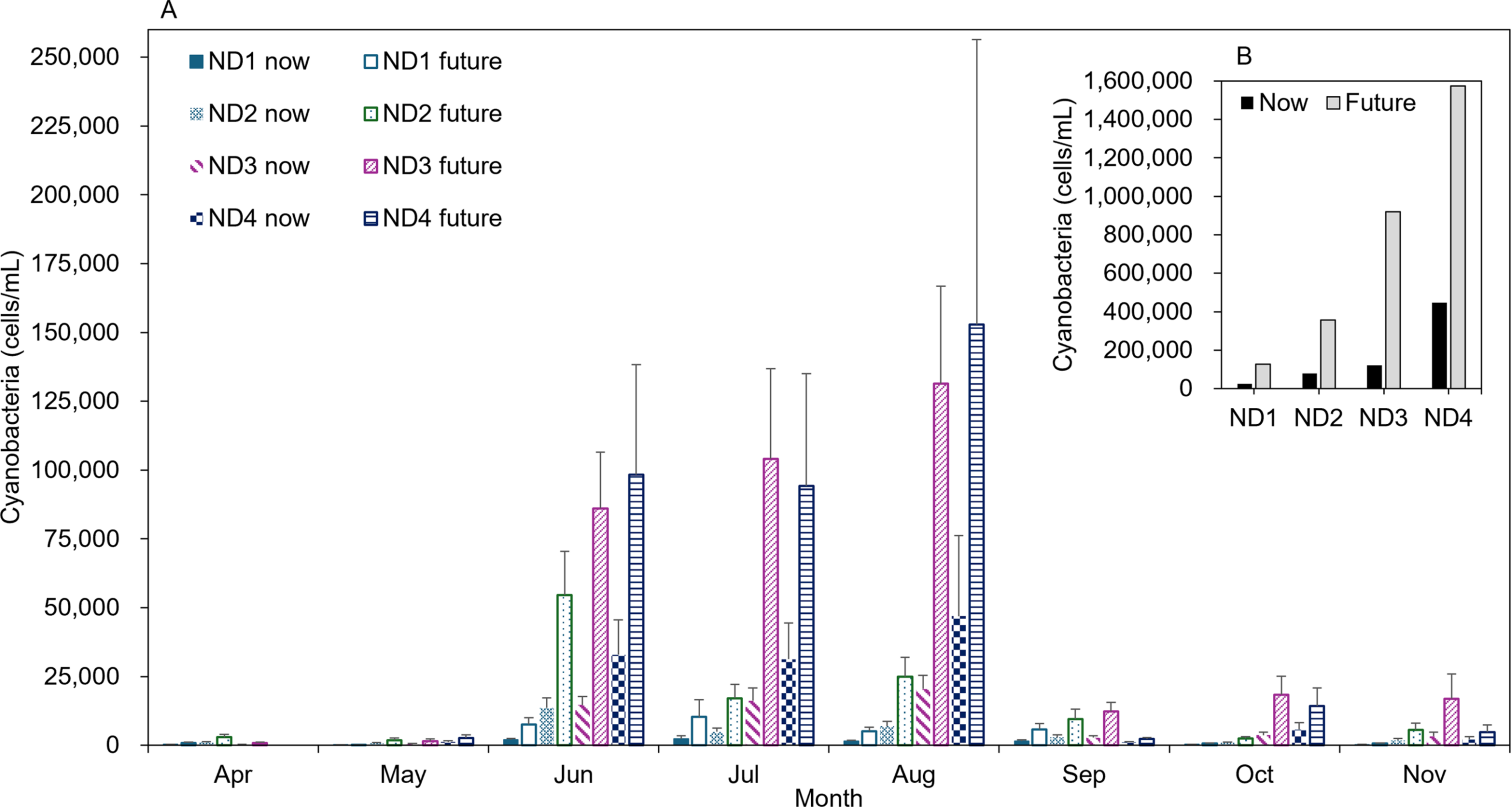
Current (“now,” predictions using observed data in 2016–2023) and climate change‐predicted (“future,” predictions using elevated temperatures projected in 2091–2100) cyanobacteria abundance: (A) monthly mean and (B) maximum cyanobacteria abundance at the study sites. The upper error bars denote standard errors.

Using the trained models, future cyanobacteria densities were predicted by inputting forecasted temperature increases (Section 2.2). The mean HCB magnitude increased at all study sites (Figure 5A). In particular, the mean cyanobacteria density from June to August was predicted to increase three to four times in the future under SSP5–8.5 scenario compared to current levels. The maximum intensity of future HCBs was simulated to exceed 1 million cyanobacteria cells/mL (Figure 5B), which is the highest alert level threshold for massive blooms in Korea, a level that has not yet been observed. The likelihood of such events occurring in ND4 is estimated to total 3.5 weeks over a 10‐year period. Furthermore, monthly cyanobacteria variation was predicted to increase drastically in the future with increased temperatures. Specifically, the largest increases in the coefficient of variation were observed from 2.64 to 2.92 at ND1 (July), from 1.42 to 1.60 at ND2 (August), from 1.16 to 1.35 at ND3 (June), and from 2.42 to 2.62 at ND4 (August). These findings underscore the need for local governments to prepare for large‐scale cyanobacteria blooms in the coming years should efforts to reduce carbon dioxide emissions fail to be widely adopted. The largest percent increase in cell density was observed at site ND3, with a 505% rise, compared to increases of 286%, 268%, and 206% at ND1, ND2, and ND4, respectively. This suggests that locations currently experiencing moderate to severe HCBs are more susceptible to climate change, potentially leading to more intense HCBs at these sites in the future.

### 3.3 Determination of PAC Dose for Removing T&O Compounds and MCs

The PAC dose required for managing specific cyanobacteria levels first required converting cyanobacteria densities into geosmin, 2‐MIB, MCs, and MC‐LR concentrations. By utilizing data from literature and the Nakdong River, the following equations were derived for calculating the concentrations of geosmin and 2‐MIB (ng/L; dissolved):

Tsao et al. (2014)

(1) $$\text{Geosmin}=10^{0.63\times \log_{10}(D_{Doli})-0.26}$$

This study

(2) $$\text{Geosmin}=0.0332\times D_{Doli}+15.48$$

Chiu et al. (2016)

(3) $$2‐\mathrm{MIB}=10^{0.508\times \log_{10}\left(G_{DP}\right)-0.6259}$$

where $D_{Doli}$ is the density of *Dolichospermum* (cells/mL), and $G_{DP}$ is the gene concentration of *Dolichospermum* and *Planktothrix* (copies/mL), calculated as: $\begin{array}{c} G_{DP}=1.95\times \text{Dolichospermum}\;\text{and}\;\; \end{array}\begin{array}{c} \text{Planktothrix}\;\left(\text{cells}/\mathrm{mL}\right)+2 \end{array}$ (Chiu et al. 2017).

This study

(4) $$2‐\mathrm{MIB}=2.5431\times \text{cyanobacteria}\;{\left(\text{cells}/\mathrm{mL}\right)}^{0.15}$$

Averages of Equations (1 and 2) (geosmin) and (3) and (4) (2‐MIB) were used to estimate concentrations from cyanobacteria counts. For the conversion of cyanobacteria cell counts to MCs concentrations, a relationship of 50,000 cyanobacteria cells/mL equating to 10 μg/L of cellular content MCs (Fitzgerald et al. 1999; WHO 2003; Health Canada 2022) was used. A correlation between *Microcystis* and MC‐LR was established based on Nakdong River data, where MC‐LR (μg/L, intracellular and extracellular) equals 0.0000126 × *Microcystis* cells/mL (*R* ^2^ = 0.629). This correlation was applied to convert cyanobacterial densities into corresponding MC‐LR concentrations. To account for dissolved (extracellular) MCs and MC‐LR, we used 80% of the calculated MCs and MC‐LR values to represent the dissolved fraction (He and Wert 2016).

To determine the required PAC dosage for certain concentrations of T&O compounds (geosmin and 2‐MIB), MCs, and MC‐LR, three empirical models were derived using data on PAC dose versus geosmin, 2‐MIB, MCs, and MC‐LR residual from earlier studies (Table S2, Figure S3). Results indicated that MCs and T&O compounds were effectively removed by using larger amounts of PAC as well as higher‐performance PAC. Additionally, the results suggest that PAC dosage required to achieve removal to a desired threshold should be increased proportionally as the initial concentration of the respective compound increases, or the dosage should be increased exponentially based on the desired target removal. For example, achieving a reduction of MCs from 10 to 1.6 μg/L (in line with EPA guidelines, 84% removal) requires 20–52 mg/L of PAC. In comparison, removing MCs from a starting concentration of 100 μg/L to the same threshold (98.4% removal) requires 43–100 mg/L of PAC. This represents an exponential increase from 10–33 mg PAC/L needed for a 68% removal, as modeled by the exponential fit to the Ho model (Figure S3; $\mathrm{PAC}\;\left(\mathrm{mg}/L\right)=0.8174e^{4.4972\times \text{removal}}$).

**FIGURE 6:**
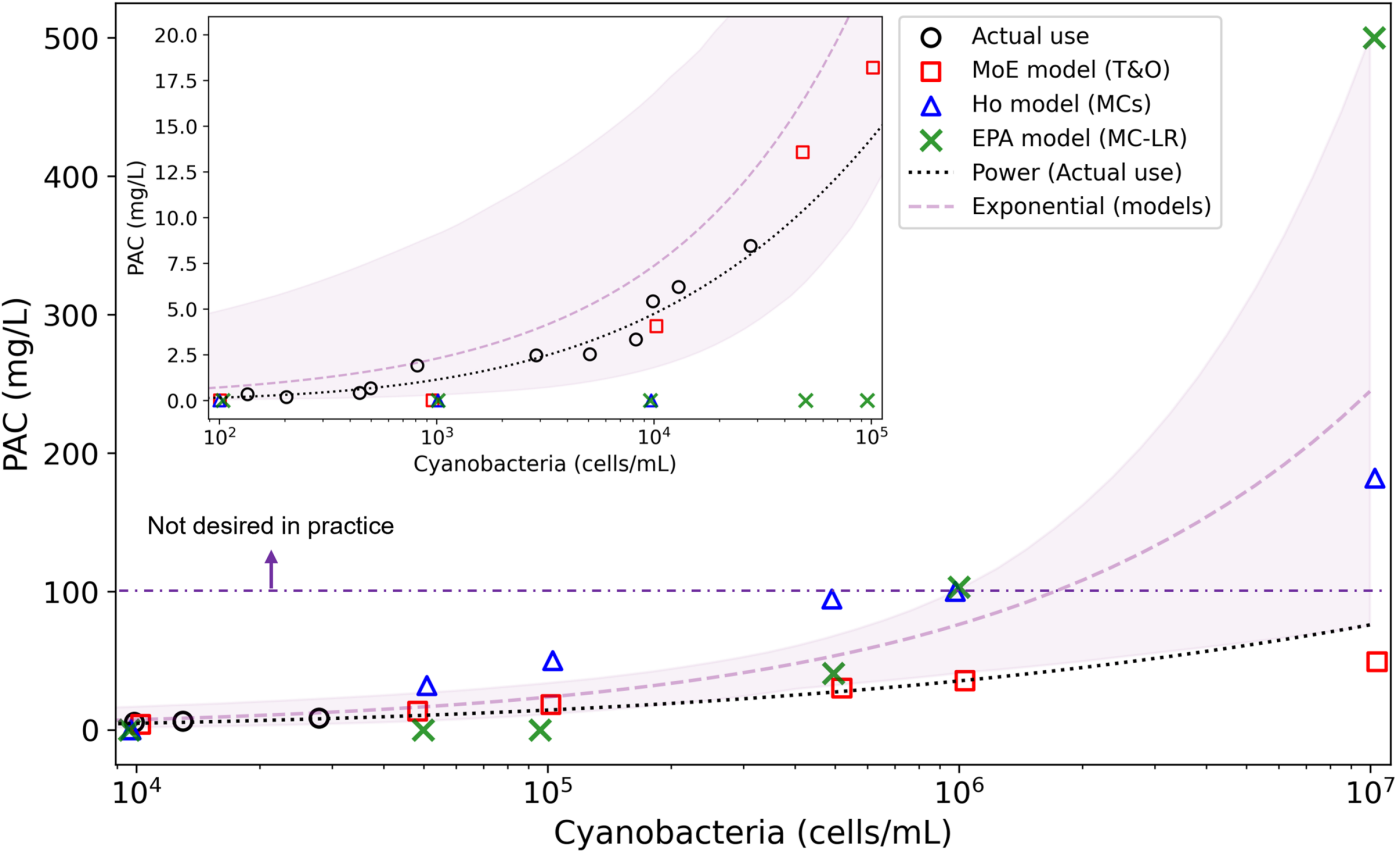
Estimation of required powdered activated carbon (PAC) dose in response to cyanobacteria levels based on three empirical models (the MoE, Ho, and EPA models) and the historical data in the water treatment plant study sites (“actual use”). “Power (actual use)” denotes the power function curve fitted to “actual use”: $y=0.0049x^{4.9577}$, *R* ^2^ = 0.954. “Exponential (models)” denotes the exponential function curve fitted to the three models: $y=0.0688e^{1.1680x}$, *R* ^2^ = 0.590. In both equations, *y* and *x* represent PAC (mg/L) and log₁₀(cyanobacteria density [cells/mL]), respectively. The shaded area indicates the 95% confidence interval to the exponential model. The dash‐dotted line notes unrealistically high PAC concentrations exceeding 100 mg/L.

Figure 6 illustrates the PAC‐dose models versus HCB levels and compares modeled PAC use to WTP observations. PAC dosage increased proportional to the fifth power of the logarithm of cyanobacteria and exponentially with the logarithmic increase in cyanobacteria levels (resulting in higher MCs and T&O levels in the source water) to meet water quality guidelines. A power law fit applied to the historical data yielded results closely aligning with those produced by the models, particularly the MoE model. However, when cyanobacteria densities exceeded 100,000 cells/mL, modeled PAC doses began to diverge. This implies that the removal efficiency of T&O compounds and MCs by PAC treatment should be precisely monitored at WTPs during severe HCBs. In the Ho and EPA models, PAC was unnecessary at low cell densities because toxins remained below guideline thresholds (1.6 μg/L for MCs; 1.0 μg/L for MC‐LR). The maximum recorded values in the country were 484 ng/L (geosmin), 140 ng/L (2‐MIB), and 41.9 μg/L (MC‐LR) in the Nakdong River, respectively. During such rare extremes, considerably higher PAC is required than the typical dosage (e.g., over 20 mg/L). Without this increased PAC dosage, some T&O compounds and MC‐LR may persist in tap water. Notably, the extreme MC‐LR event that occurred in 2022 coincided with reports from non‐governmental organizations (Yang 2022; Ko 2023) indicating that MCs were detected in tap water sourced from the Nakdong River, suggesting a possible correlation. When possible, attention should be given to monitoring the presence of negatively charged MCs, such as MC‐LR, during severe HCBs because the treatment of such MCs requires more PAC or a longer contact time than neutrally charged MCs (Newcombe 2006; Huang and Lenhart 2024).

Estimated PAC doses at very high cyanobacteria densities are uncertain. Nevertheless, previous laboratory research sometimes includes results with PAC doses greater than 50 mg/L for adsorbing MCs (Cook and Newcombe 2002; Drogui et al. 2012; Zhu et al. 2016), and approximately 50 mg/L for T&O removal (Cook et al. 2001), corresponding densities approximating 10^6^ cells/mL. These dosages align with the findings of this study and provide support for the model results depicted in Figure 6. Although PAC dose exceeding 100 mg/L is impractical for WTPs (sludge management and filtering issues), this study presents the quantitative PAC‐increase profile versus HCB magnitude. In the event of extreme HCBs (i.e., cyanobacteria exceeding 1,000,000 cells/mL), adopting longer contact time (> 1 h) and additional processes (e.g., ozonation or granular activated carbon (GAC)) may be needed.

### 3.4 Estimation of PAC Usage and Cost in the Future According to Climate Change Scenario

Based on the findings in previous sections, the cost of using PAC for the treatment of cyanobacteria in water to meet the guideline levels of T&O compounds, MCs, and MC‐LR (Section 2.3) by 2100 was estimated (Table 1). The costs were estimated under two scenarios: a low‐emission case in which current HCB patterns persist (best case), and SSP5–8.5, which projects larger HCB severity with rising temperatures (worst case). Sludge‐treatment cost was included by converting PAC use to equivalent sludge quantities (Crittenden et al. 2022). K‐water WTPs typically spend $78.1 per ton to treat sludge, which is subsequently used as raw material for cement, as well as fill or cover materials.

**TABLE 1:** Estimated dosage and cost of powdered activated carbon (PAC) for treatment of taste and odor compounds, microcystins, and microcystin‐LR, corresponding to projected cyanobacteria levels under best‐ and worst‐case scenarios at the Nakdong River study sites. Data in June, July, August, and September were used.

	Water source site	Scenario	Cyanobacteria in raw water (cells/mL)	Theoretical mean PAC dose (mg/L)	Theoretical mean required PAC (kg, monthly)	Cost required for purchasing PAC (monthly)	Cost burden in the future^a^ (per household per month)
Average^b^		Best	20,094	10.5	1,527,542	$2,403,776	$5.25
		Worst	67,399	19.4	2,822,248	$4,441,155	$9.70
Mean of > 75% ile^c^	ND1	Best	5659	5.5	67,913	$106,870	$2.78
		Worst	24,381	11.6	142,474	$224,200	$5.83
	ND2	Best	22,880	11.2	336,385	$529,344	$5.62
		Worst	90,187	22.4	674,540	$1,061,473	$11.25
	ND3	Best	43,379	15.5	219,372	$345,209	$7.71
		Worst	284,055	40.2	569,073	$895,507	$20.23
	ND4	Best	107,851	24.6	2,192,597	$3,450,322	$12.29
		Worst	342,232	44.1	3,938,635	$6,197,927	$22.07
Mean of ≤ 50% ile^c^	ND1	Best	327	1.3	15,979	$25,145	$0.65
		Worst	859	2.1	26,095	$41,065	$1.07
	ND2	Best	685	1.9	56,751	$89,304	$0.94
		Worst	1271	2.6	77,646	$122,186	$1.29
	ND3	Best	1010	2.3	32,579	$51,266	$1.15
		Worst	3597	4.4	62,040	$97,628	$2.20
	ND4	Best	1006	2.3	204,303	$321,496	$1.14
		Worst	2010	3.3	290,842	$457,629	$1.63

^a^ Value including sludge treatment cost and adjusted for inflation at an anticipated total rate of 630.8% anticipated at year 2100.

^b^ Weighted monthly mean for the four sites on the Nakdong River considering different water treatment volumes. Total water treatment capacity of the four sites is 4,753,100 cubic meters per day.

^c^ Selected cyanobacteria data at each site were used.

Required PAC varied substantially by location and scenario: under SSP5–8.5, 2100 monthly household costs range from $5.8 (ND1) to $22.1 (ND4); when cyanobacteria densities are ≤ median the costs are $1.1–$2.2, rising to $5.8–$22.1 (ND1–ND4) under the worst case (Table 1). The PAC doses used for the cost estimation fell within the typical range of up to 60 mg/L (Bernal‐Romero del Hombre Bueno et al. 2019), suggesting that the estimations in this study are reasonable.

Currently, average PAC cost during a one‐year bloom season at the study sites is estimated at $2.9–5.3 per household (Table 1), consistent with inflation‐adjusted earlier estimates ($6.0–8.3/household/year; Lee et al. 2008). Under SSP5–8.5, mean monthly costs in 2100 across the Nakdong River are projected at $9.7 per household (≈ $1.3 in present‐day value). Accounting for inflation, this represents 650% of the previously estimated current expenditure of $0.2 per household per month (Figure 3), which may be too burdensome for an average household to afford. This increase exceeds previously reported willingness‐to‐pay increases of 46%–200% on water bills for safe drinking water (Vásquez et al. 2009; Vásquez 2014). Because lower thresholds are recommended for children (e.g., 0.3 μg/L MCs for under‐six; US EPA 2015) and new water quality standards are being established (e.g., per‐ and polyfluoroalkyl substances, PFAS) (Murray et al. 2019; Sadia et al. 2023; US EPA 2024), future treatment costs are likely to rise further. Therefore, it is essential to prepare in advance by forecasting and implementing proactive measures to operate WTPs efficiently and mitigate HCBs.

**FIGURE 7:**
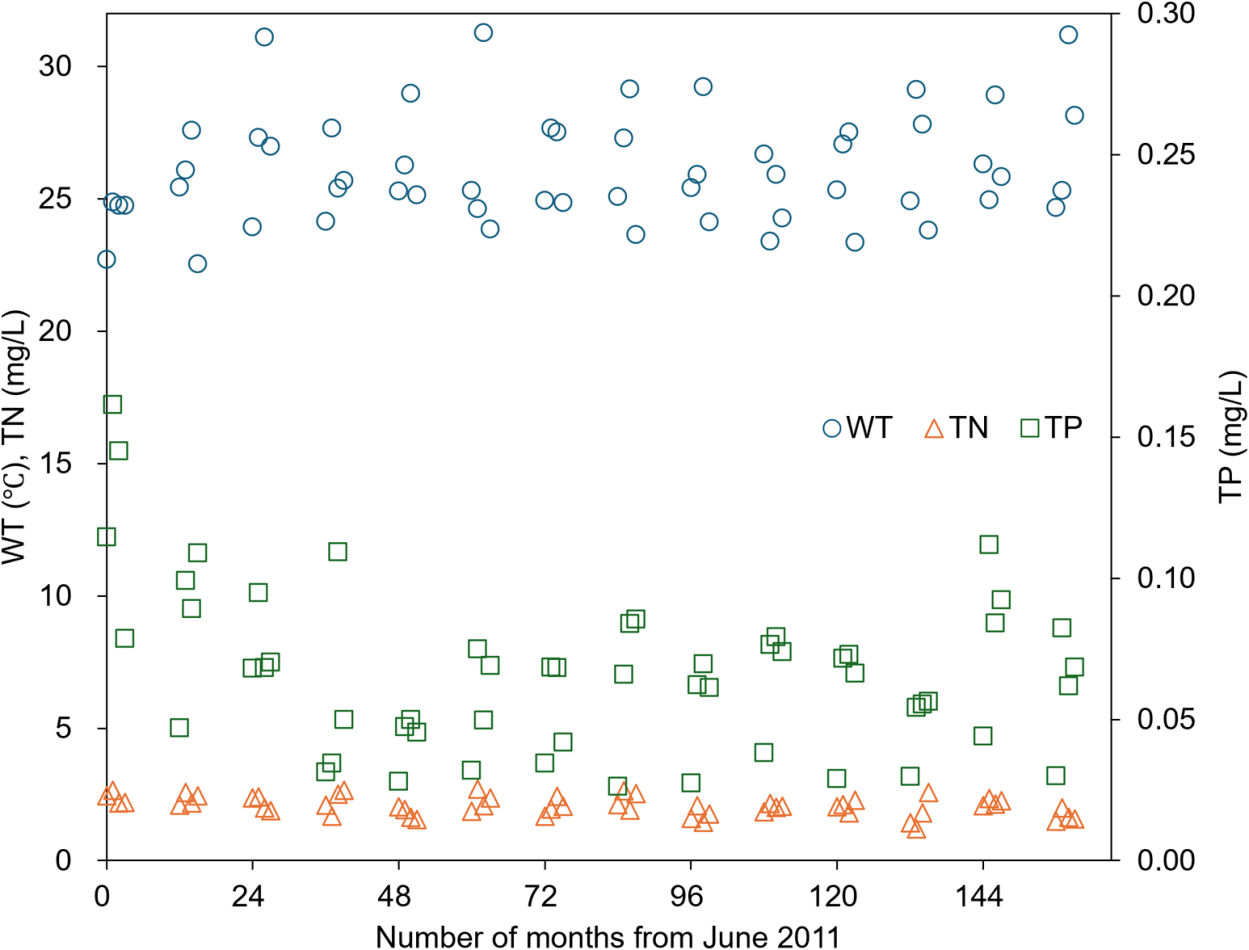
Time‐course of water temperature (WT), total nitrogen (TN), and total phosphorus (TP) in the Nakdong River. Values represent the monthly means for June, July, August, and September across the four study sites. Notably, stricter TP regulations for wastewater treatment facility discharges were implemented in 2012. A Mann–Kendall test on the 14‐year series indicates a positive temperature tendency (*τ* = 0.297; Sen's slope = 0.0854°C/yr.; *p* = 0.155), a decline in TN (*τ* = −0.473; slope = −0.0376 mg/L/yr.; *p* = 0.021), and no significant trend in TP (*τ* = −0.165; slope = −0.0012 mg/L/yr.; *p* = 0.443).

The observed upward trend in water temperature over the past 14 years supports the appropriateness of using elevated temperatures to simulate future conditions in this study (Figure 7). To mitigate rising and fluctuating water treatment costs, nutrient levels could be managed at pollution sources, especially given the projected rise of nutrient levels in the future (Huisman et al. 2018). Management efforts could focus on maintaining TP levels under 0.05 mg/L and TN under 1.8 mg/L (Shan et al. 2020). Additionally, as PAC demand increases, life‐cycle costs from other chemicals and treatment processes, and associated energy and emissions, should be considered. With water quality projected to deteriorate due to climate change (Mi et al. 2019), a previous analysis indicated a significant potential increase in drinking water production costs. These costs could increase from $2.0 to $11.3 per month per household in the worst case, driven primarily by higher sludge treatment and disposal costs resulting from residual chemicals linked to water‐quality deterioration (Verlicchi et al. 2024). This relative increase represents a 465% escalation, which is comparable to the trends observed in this study.

A limitation is that predictions considered temperature changes only, assuming other factors such as nutrients remain constant. This assumption is based on evidence that temperature is a major factor influencing the proliferation of cyanobacteria (O'Neil et al. 2012; Mowe et al. 2015), which was verified using RF models in this study. Climate‐driven increases in precipitation intensity can enhance nutrient inputs via runoff, thereby favoring short‐term bloom dynamics. Scenarios explicitly varying future nutrient inputs were not modeled because long‐term nutrient trajectories are highly uncertain. Notably, the 14‐year monthly record shows no long‐term TN or TP increase comparable to the temperature tendency (Figure 7). Unlike temperature, nutrient loads are strongly influenced by management and policy (e.g., the TP decrease observed in 2011–2012; Figure 7), and may therefore increase or decrease depending on future socio‐political and technological pathways. Nutrient levels can be altered by anthropogenic actions and regulations. Furthermore, converting cyanobacteria density to the concentrations of T&O compounds and MC‐LR and assigning PAC doses also does not capture all environmental variability or differences among PAC types. Additionally, more data (e.g., up to 10 years) may be required to more reliably generalize the model and predict long‐term HCB patterns. Nevertheless, predicting T&O compounds and MC‐LR concentrations would not have been feasible without the methodology adopted in this study.

Further studies could expand the presented framework to account for other processes. For example, it would be important to explore variations in HCB occurrences as a function of both temperature and extreme meteorological events, such as the frequency and magnitude of intense precipitation. It is crucial to develop efficient treatment strategies in response to the increasing frequency and high variability of HCB events. This may include constructing additional treatment facilities and employing alternative treatment chemicals or processes. Moreover, one outcome of this study is that WTP operators should consider planning for modified treatment strategies to account for future scenarios with more frequent HCBs. Strategies include adopting alternative treatment methods and adjusting daily source‐water intake volumes. The annualized capital cost for ozonation and GAC facilities is approximately $20/household/year (estimated in this study based on Guo et al. 2014; Hilbig et al. 2020; HDR Inc. 2022). Therefore, regions projected to experience frequent HCBs with cyanobacteria cell counts exceeding 500,000 cells/mL (≈40 mg/L PAC; Figure 6) should consider adopting ozonation and GAC treatment, because sustained high‐dose PAC application is operationally challenging and potentially less cost‐effective than investing in these facilities. Most importantly, global efforts should aim to prevent SSP5–8.5, the worst climate change scenario, from becoming a reality by adopting net‐zero policies.

## 4 Conclusions

To our knowledge, this study is the first to estimate the impact of climate change on surface water in relation to HCBs and water treatment operations. Historical operational data from WTP study sites indicated a correlation between the usage of PAC and HCB occurrences. RF modeling, leveraging cumulative data from the Nakdong River study sites, revealed substantial increases in both the magnitude (400,000 versus 1,500,000 cells/mL) and variability of HCBs in response to the SSP5–8.5 scenario by the year 2100. Given the correlation between *Dolichospermum* and *Planktothrix* with T&O compounds, and *Microcystis* with MC‐LR, substantially higher PAC dosages were required to treat elevated cyanobacterial densities. Finally, cost estimates for treating T&O compounds and MCs in response to rising cyanobacteria levels suggest that the cost burden in a household could reach up to $22.1/month in 2100, with a minimum of $1.1 at the same site (excluding winter). The largest increase in maximum burden was between $7.7 (if current HCBs continue until 2100) and $20.2 (under the worst‐case temperature rise scenario) per month per household. Water treatment costs could be even higher due to the use of other chemicals, supply chain inefficiencies, and implementation of more stringent drinking water quality standards. Thus, these findings underscore the urgent need to manage surface water quality and achieve net‐zero emissions while enhancing preparedness for increasingly complex HCB occurrence patterns in the future. At some sites, adopting ozone/GAC facilities may be a more efficient long‐term strategy. Intelligent water use and treatment management strategies must be implemented to minimize the risks to assets, resources, and public health. Further studies should evaluate treatment costs more comprehensively by incorporating various environmental conditions along with the employment of additional chemicals, disinfection processes, and ozonation techniques. This could offer deeper insights into managing HCBs and other contaminants, including DBPs and PFAS.

## Author Contributions

**Jayun Kim:** data curation, formal analysis, methodology, validation, visualization, conceptualization, software, supervision, writing – original draft, writing – review and editing. **Himchan Park:** data curation, formal analysis, methodology, validation, visualization, writing – original draft. **John J. Lenhart:** supervision, validation, investigation, resources, writing – review and editing. **Jiyoung Lee:** supervision, validation, investigation, resources, writing – review and editing. **Kendall Byrd:** investigation. **Gayeon Jang:** project administration, resources. **Sangjun Kim:** methodology. **Joonhong Park:** funding acquisition, investigation.

## Funding

This study was supported by the Basic Science Research Program through the National Research Foundation of Korea, funded by the Ministry of Education (No. 2018R1A6A1A08025348) and the Korea Environment Industry & Technology Institute through the project for developing innovative drinking water and wastewater technologies program funded by the Korean Ministry of the Environment (2020002700003). Additional support was provided by the Ohio Water Development Authority through Award #10,033.

## Ethics Statement

The authors have nothing to report.

## Consent

All authors have reviewed and approved this manuscript and give their consent for its publication.

## Conflicts of Interest

The authors declare no conflicts of interest.

## Supporting information


**Table S1:** Empirical equations for flow rate and water velocity developed for study sites along the Nakdong River to determine water velocity values.
**Table S2:** Conditions for the powdered activated carbon (PAC) dose tests in previous studies which data were used to estimate PAC dose for removing taste and odor compounds, total microcystins, and microcystin‐LR.
**Figure S1:** Water intake sources in South Korea. “Reservoir” refers to water stored behind dams on lakes or rivers. “Other reservoirs” include naturally formed lakes, relatively small and originally used for agricultural purposes.
**Figure S2:** Feature importance for random forest predictions of cyanobacteria at the study sites. WT, water temperature (°C); TN, total nitrogen (mg/L); DO, dissolved oxygen (mg/L); Chl‐a, chlorophyll‐a (mg/m^3^); Vel, water velocity (m/s); EC, electrical conductivity (μS/cm); TP, total phosphorus (mg/L); SS, suspended solids (mg/L); COD, chemical oxygen demand (mg/L); BOD, biochemical oxygen demand (mg/L).
**Figure S3:** Derived models for estimating powdered activated carbon (PAC) dose for a certain amount of (A) microcystins (MCs, the Ho model), (B) taste and odor compounds (data for geosmin and 2‐MIB were averaged, the MoE model), (C) and MC‐LR (the EPA model). Refer to Table S2 for detailed information on the conditions of the models.

## Data Availability

The data that support the findings of this study are available from the corresponding author upon reasonable request.
