# Supplementary material for: Estimating Operational Costs of Activated Carbon for Water Treatment Plants by Predicting the Rise of Harmful Algal Blooms Under Climate Change in Korea Using Machine Learning
Source: Water Environ Res. 2026 Mar 10;98(3):e70310. doi: 10.1002/wer.70310 (PMC12976194; doi:10.1002/wer.70310)
Supplement: Supplementary file 1 — Table S1: Empirical equations for flow rate and water velocity developed for study sites along the Nakdong River to determine water velocity values. Table S2: Conditions for the powdered activated carbon (PAC) dose tests in previous studies which data were used to estimate PAC dose for removing taste and odor compounds, total microcystins, and microcystin‐LR. Figure S1: Water intake sources in South Korea. “Reservoir” refers to water stored behind dams on lakes or rivers. “Other reservoirs” include naturally formed lakes, relatively small and originally used for agricultural purposes. Figure S2: Feature importance for random forest predictions of cyanobacteria at the study sites. WT, water temperature (°C); TN, total nitrogen (mg/L); DO, dissolved oxygen (mg/L); Chl‐a, chlorophyll‐a (mg/m3); Vel, water velocity (m/s); EC, electrical conductivity (μS/cm); TP, total phosphorus (mg/L); SS, suspended solids (mg/L); COD, chemical oxygen demand (mg/L); BOD, biochemical oxygen demand (mg/L). Figure S3: Derived models for estimating powdered activated carbon (PAC) dose for a certain amount of (A) microcystins (MCs, the Ho model), (B) taste and odor compounds (data for geosmin and 2‐MIB were averaged, the MoE model), (C) and MC‐LR (the EPA model). Refer to Table S2 for detailed information on the conditions of the models. [file WER-98-e70310-s001.docx]

Supporting Information

**Estimating operational costs of activated carbon for water treatment plants by predicting the rise of harmful algal blooms under climate change in Korea using machine learning**

Jayun Kim ^1,2^, Himchan Park ^1,3^, John J. Lenhart ^4^, Jiyoung Lee ^2,5^, Kendall Byrd ^2^, Gayeon Jang ^1^, Sangjun Kim ^1^, Joonhong Park ^1^

^1^Department of Civil and Environmental Engineering, Yonsei University, Seoul 03722, The Republic of Korea

^2^Division of Environmental Health Sciences, College of Public Health, The Ohio State University, Columbus, OH 43210, USA

^3^Department of Intelligent Data and Optimization, Yonsei University, Seoul 03722, The Republic of Korea

^4^Department of Civil, Environmental and Geodetic Engineering, The Ohio State University, Columbus, OH 43210, USA

^5^Department of Food Science & Technology, The Ohio State University, Columbus, OH 43210, USA

Correspondence: Jayun Kim (nature9973@gmail.com)

Jayun Kim and Himchan Park contributed equally as first authors.

Table S1. Empirical equations for flow rate and water velocity developed for study sites along the Nakdong River to determine water velocity values.

| Site | Flow rate ($Q$, m^3^/s)-water velocity ($v$, m/s) relationship | Reference |
| --- | --- | --- |
| ND1 | $v=0.0004\times Q-0.0011$, *R*^2^ = 0.977 | Kim et al. (2026) |
| ND2 | $v=0.0003\times Q+0.0005$, *R* ^2^ = 0.950 |  |
| ND3 | $v=-2\times{10}^{-8}\times Q^{2}+0.0004\times Q$, *R* ^2^ = 0.994 |  |
| ND4 | $v=-2\times{10}^{-8}\times Q^{2}+0.0003\times Q$, *R* ^2^ = 0.999 |  |

Table S2. Conditions for the powdered activated carbon (PAC) dose tests in previous studies which data were used to estimate PAC dose for removing taste and odor compounds, total microcystins, and microcystin-LR.

| Model | Target | Initial concentration | Contact time | Background water | PAC specifications |
| --- | --- | --- | --- | --- | --- |
| MoE | Geosmin and 2-MIB | 100 ng/L | 30 min | Raw surface water  UV_254_ = 0.0650 /cm | Iodine adsorption capacities = 1089 and 1087 mg/g  Methylene Blue decolorization capacities = 220 and 190 mL/g  (PAC-1 and PAC-2)  External surface area ≥ 600 m^2^/g  Pore volume ≥ 1.2 cc/g  Average pore diameter ≥ 40 Å |
| Ho | Microcystins | 4 µg/L each of MC-RR, MC-YR, and MC-LA, 10 µg/L of MC-LR, and  20 µg/L of cylindrospermopsin | 30 min | Dissolved organic carbon = 5.0 and 4.3 mg/L  UV_254_ = 0.093 and 0.076 /cm  pH = 7.5 and 7.6 | Starting material: coal. Method of activation: steam. Effective size (µm): 20–25 (PAC-A) and 10 (PAC-B). BET surface area (m^2^/g): 1289 (PAC-A) and 1105 (PAC-B). Bulk density (g/cm^3^): 0.35–0.45 (PAC-A) and 0.30–0.40 (PAC-B). |
| US EPA | Microcystin-LR | 62.5 µg/L | 1 h | Raw surface water | Not specified. Similar to PAC used in Bajracharya et al. (2019): Bituminous coal, activation method: steam, size distribution (µm) = 2–56, BET surface area (m^2^/g) = 950, total pore volume = 0.56 cm^3^/g, mesopore volume = 0.15 cm^3^/g |


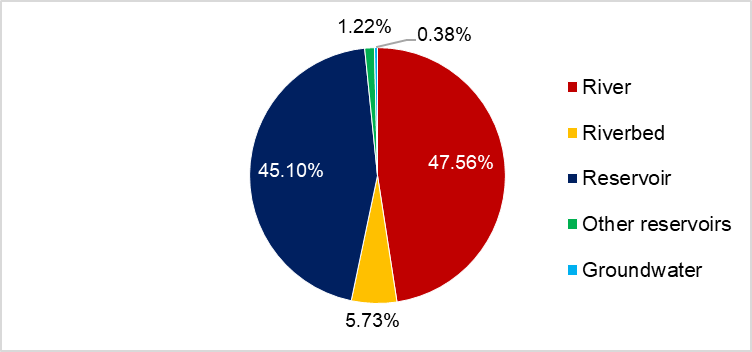


Figure S1. Water intake sources in South Korea excluding Jeju Island. “Reservoir” refers to water stored behind dams on lakes or rivers. “Other reservoirs” include naturally formed lakes, relatively small and originally used for agricultural purposes.


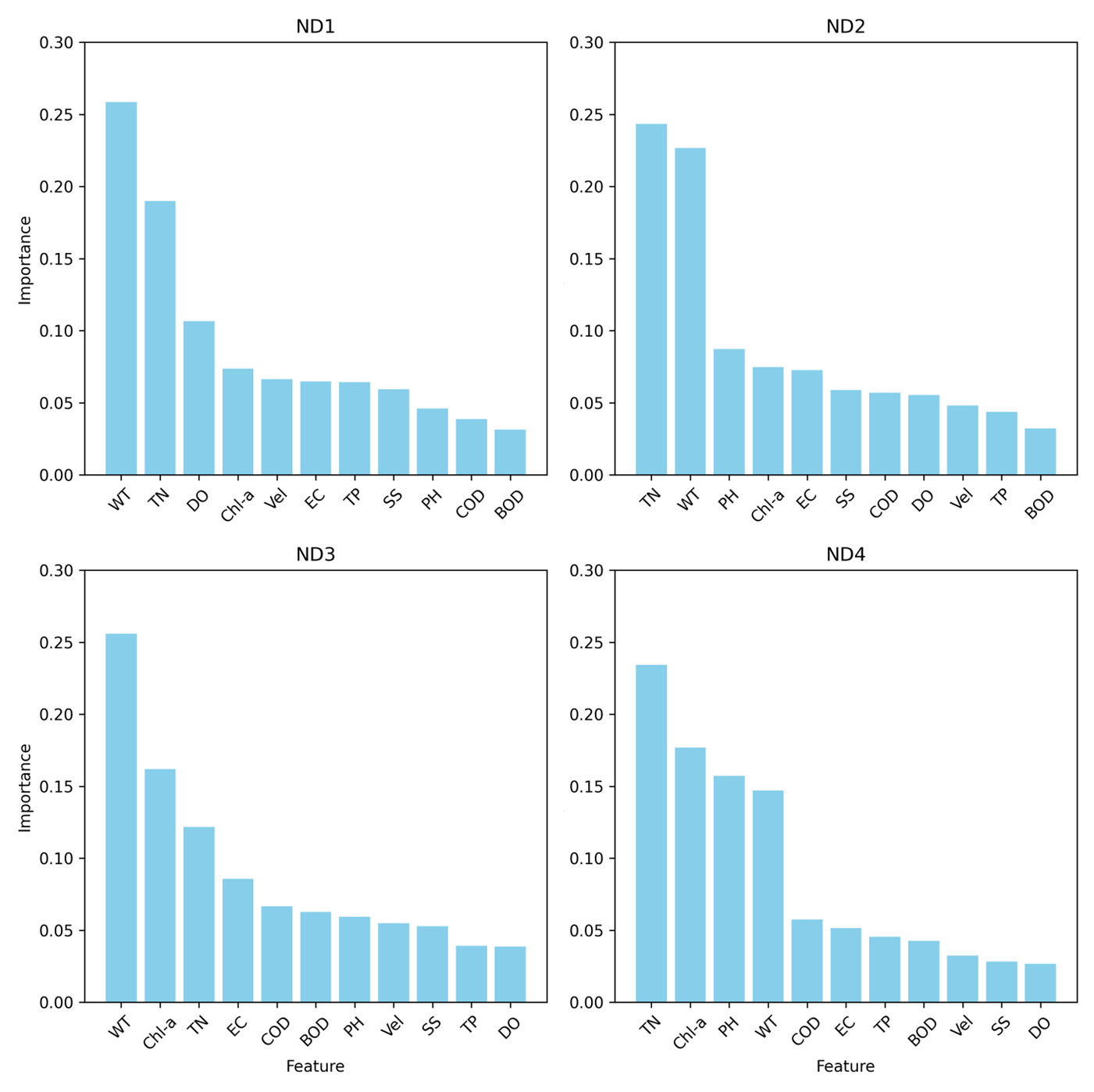


Figure S2. Feature importance for random forest predictions of cyanobacteria at the study sites. WT, water temperature (℃); TN, total nitrogen (mg/L); DO, dissolved oxygen (mg/L); Chl-a, chlorophyll-a (mg/m^3^); Vel, water velocity (m/s); EC, electrical conductivity (μS/cm); TP, total phosphorus (mg/L); SS, suspended solids (mg/L); COD, chemical oxygen demand (mg/L); BOD, biochemical oxygen demand (mg/L).


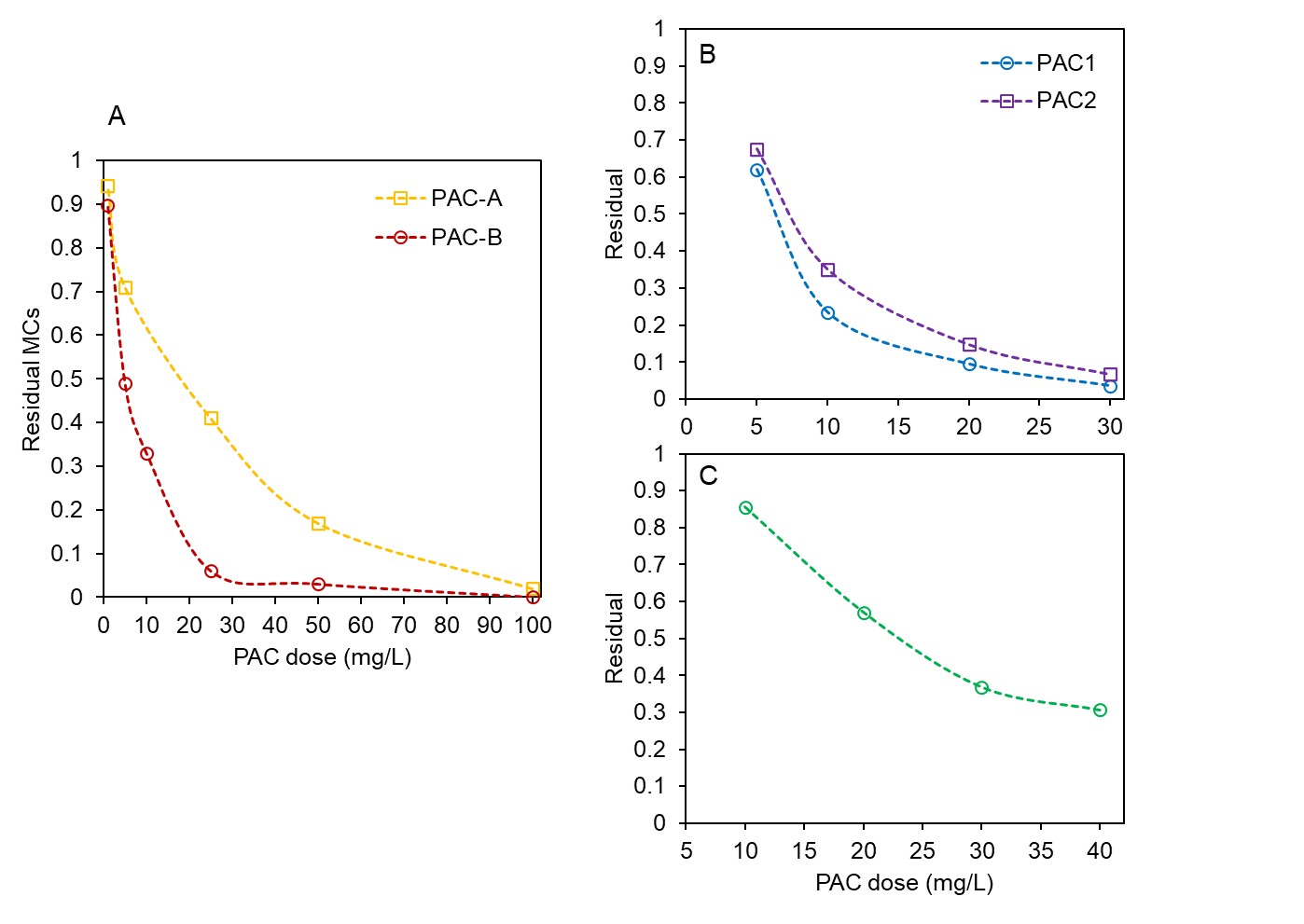


Figure S3. Derived models for estimating powdered activated carbon (PAC) dose for a certain amount of (A) microcystins (MCs, the Ho model), (B) taste and odor compounds (data for geosmin and 2-MIB were averaged, the MoE model), (C) and MC-LR (the EPA model). Refer to Table S2 for detailed information on the conditions of the models.

References

Bajracharya, A., Liu, Y.-L., & Lenhart, J.J. (2019). The influence of natural organic matter on the adsorption of microcystin-LR by powdered activated carbon. *Environmental Science: Water Research & Technology*, *5*, 256–267. https://doi.org/10.1039/C8EW00582F

Kim, J., Jang, G., Jo, M., & Park, J. (2026). Simulation of harmful cyanobacterial blooms under weir operations and climate change scenarios: A data-driven study of the Nakdong River. *Environmental Engineering Research*, *31*(1), 250081. https://doi.org/10.4491/eer.2025.081
